# Quality indicators for care of depression in primary care settings: a systematic review

**DOI:** 10.1186/s13643-017-0530-7

**Published:** 2017-07-03

**Authors:** Yelena Petrosyan, Yeva Sahakyan, Jan M. Barnsley, Kerry Kuluski, Barbara Liu, Walter P. Wodchis

**Affiliations:** 1 Institute of Health Policy, Management and Evaluation, University of Toronto, 155 College Street, 4th Floor, Toronto, Ontario M5T 3M6 Canada; 2 Toronto Health Economics and Technology Assessment (THETA) Collaborative, Toronto General Hospital Research Institute, University Health Network, 200 Elizabeth Street, 10th Floor, Toronto, Ontario M5G 2C4 Canada; 3 Dalla Lana School of Public Health, University of Toronto, 155 College Street, 4th Floor, Toronto, M5T 3M6 Ontario Canada; 4 Lunenfeld Tanenbaum Research Institute, Sinai Health System, 1 Bridgepoint Drive, 14 St. Matthews Road, Toronto, Ontario M4M 2B5 Canada; 5 Sunnybrook Health Sciences Centre, University of Toronto, 2075 Bayview Ave., Room H4 79, Toronto, ON M4N 3M5 Canada; 6 Institute for Clinical Evaluative Sciences, Toronto, Canada; 7 Toronto Rehabilitation Institute, Toronto, Canada

**Keywords:** Quality indicators, Major depression, Primary care, Quality of care, Quality assessment, Quality monitoring

## Abstract

**Background:**

Despite the growing interest in assessing the quality of care for depression, there is little evidence to support measurement of the quality of primary care for depression. This study identified evidence-based quality indicators for monitoring, evaluating and improving the quality of care for depression in primary care settings.

**Methods:**

Ovid MEDLINE and Ovid PsycINFO databases, and grey literature, including relevant organizational websites, were searched from 2000 to 2015. Two reviewers independently selected studies if (1) the study methodology combined a systematic literature search with assessment of quality indicators by an expert panel and (2) quality indicators were applicable to assessment of care for adults with depression in primary care settings. Included studies were appraised using the Appraisal of Indicators through Research and Evaluation (AIRE) instrument, which contains four domains and 20 items. A narrative synthesis was used to combine the indicators within themes. Quality indicators applicable to care for adults with depression in primary care settings were extracted using a structured form. The extracted quality indicators were categorized according to Donabedian’s ‘structure-process-outcome’ framework.

**Results:**

The search revealed 3838 studies. Four additional publications were identified through grey literature searching. Thirty-nine articles were reviewed in detail and seven met the inclusion criteria. According to the AIRE domains, all studies were clear on purpose and stakeholder involvement, while formal endorsement and usage of indicators in practice were scarcely described. A total of 53 quality indicators were identified from the included studies, many of which overlap conceptually or in content: 15 structure, 33 process and four outcome indicators. This study identified quality indicators for evaluating primary care for depression among adult patients.

**Conclusions:**

The identified set of indicators address multiple dimensions of depression care and provide an excellent starting point for further development and use in primary care settings.

**Electronic supplementary material:**

The online version of this article (doi:10.1186/s13643-017-0530-7) contains supplementary material, which is available to authorized users.

## Background

Depression is a common mental health problem for adults associated with decreased quality of life, productivity loss, family stress, increased utilization and cost of healthcare, and all-cause mortality [1–5]. The 12-month prevalence of depression is 4.7%, with a lifetime prevalence of up to 16.6% in adults aged 18 years and older [6]. Previous research showed that the rate of depression was higher among older adults within hospitals (21%) [1] and long-term care facilities (14%) [7]. It is well established that depression in combination with a wide range of chronic conditions, including diabetes, cardiovascular disease, cancer and arthritis, can have a negative impact on the outcomes of these conditions [8–10].

Depressive disorders are most often managed by a primary care physician, unless the severity of depression is such that care is required from a specialist [11, 12]. The Institute of Medicine defines primary care as ‘the provision of integrated, accessible health care services by clinicians that are accountable for addressing a large majority of personal health care needs, developing a sustained partnership with patients, and practicing in the context of family and community’ [13].

However, poor levels of detection, treatment and monitoring of depression have been highlighted in primary care settings [14]. Research shows that only a minority of patients with a depressive or anxiety disorder are treated in a primary care setting in accordance with clinical guidelines [15, 16]. Literature suggests that people with any chronic condition frequently experience comorbid depressive symptoms that often go undetected [1, 17].

Assessment and monitoring of care quality has become crucial for healthcare systems worldwide to enhance the accountability of healthcare providers, to improve resource allocation efficiency, to identify and minimize medical errors and to improve health outcomes [11, 18]. The Institute of Medicine defines quality of health care as ‘the degree to which health services for individuals and populations increase the likelihood of desired health outcomes and are consistent with current professional knowledge’ [19].

The assessment and monitoring of care quality can be achieved by using quality indicators which are based on standards of care and the best available evidence [20]. Data generated from these measures can be used to assess past performance, identify suboptimal practices and plan improvements. Donabedian has conceptualized the assessment of quality through examining the structures, processes and outcomes of care, and many quality indicators have been classified using this framework [21, 22].

The literature suggests that quality indicators should be evidence-based and be derived from the academic literature [20]. However, when scientific evidence is lacking, quality indicators can be defined by an expert panel of professionals by means of consensus techniques based on their experience. Evidence suggests that the systematic method of combining scientific evidence and expert opinion is the most rigorous way of developing quality indicators [11, 23].

Despite the growing interest in assessing the quality of care for depression, there has been little evaluation of the quality of care for depression in primary care settings [11, 24].

This project aimed (1) to identify evidence-based and valid quality indicators feasible for monitoring, evaluating and improving the quality of care for depression among adults over 18 years in primary care settings and (2) to critically appraise a set of identified quality indicators, using the AIRE (Appraisal of Indicators through Research and Evaluation) instrument. We conducted a systematic review to identify existing quality indicators for primary care for depression both in Canada and internationally.

## Methods

### Search strategy

A systematic literature search was conducted using Ovid MEDLINE and Ovid PsycINFO databases from January 2000 to June 2015, restricted to English articles of human studies, and when the participants consisted of adults over 18 years. The search terms used combined keywords and medical subject headings for depression and quality indicators. We used the following search terms to identify studies related to quality indicator development: ‘performance indicator(s)/measure(s)’, or ‘quality indicator(s)/measure(s)’, or ‘benchmark’, or ‘report card’ or ‘quality of health care’, or ‘clinical guideline’, or ‘quality assurance’. To identify studies related to depression care, we used the following search terms: ‘depression’ or ‘depressive disorder(s)’. The results from these two search steps were then combined (see Additional file 1).

In addition, we conducted a grey literature search to find information about quality indicator development initiatives that were not published in peer-reviewed journals. For that purpose, we searched available public repositories, including the National Quality Measures Clearinghouse (NQMC; http://www.qualitymeasures.ahrq.gov) and the National Quality Forum (NQF; http://www.qualityforum.org). Additionally, we looked for existing indicators at websites of major organizations involved in quality measurement and reporting of indicators for assessing the quality of depression care, including the RAND Health Corporation/Assessing Care of Vulnerable Elders (ACOVE), Canadian Mental Health Association (www.cmha.ca) and the online inventory maintained by the Center for Quality Assessment in Mental Health (www.cqaimh.org).

### Study selection

The literature suggests the most rigorous way of developing quality indicators is through a systematic literature search combined with consensus techniques [11, 25, 26]. Where possible, quality indicators should be derived from scientific evidence [11, 26]. The stronger the evidence, the stronger the potential benefit of quality indicators in terms of increase in the likelihood of achieving the best possible clinical outcomes [11, 26]. The main reasons for developing measures using consensus techniques include synthesizing accumulated expert opinion, enhancing decision-making, facilitating development of indicators where evidence alone is insufficient and identifying areas of care where there is controversy or uncertainty [11].

Therefore, articles were included for the purpose of this study if both of the following criteria were met:

- The study methodology combined a systematic literature search/development of indicators from clinical guidelines with assessment of quality indicators by an expert panel.
- The identified quality indicators are applicable to the provision of primary care for depression among adult patients.

The identified titles were entered into a bibliographical database and duplicates were removed. One of the reviewers (YP) checked for the selected keywords in the title, abstract and subject heading of the articles. The resulting abstracts were included for full-text review. Two reviewers (YP and YS) independently conducted full-text review according to the inclusion criteria. Also, the references of selected articles were screened for other relevant studies that had not been found in the electronic search. The resulting set of articles was included in the methodological assessment process using the AIRE (Appraisal of Indicators through Research and Evaluation) instrument. The level of agreement between reviewers evaluating studies for inclusion and undertaking methodological assessments was assessed using kappa statistics [27].

Depression in this systematic review connotes major depression and dysthymia, since most clinical practice guidelines only address treatment of major depression [1]. Previous studies demonstrate that treatments for major depression also apply to dysthymic disorders [28–30]. Major depression disorder is defined ‘as a period lasting at least 2 weeks characterized either by depressed mood (most of the day, nearly every day) and/or markedly diminished interest or pleasure in all, or almost all, activities (most of the day, nearly every day), during which a patient experiences five or more symptoms; the symptoms cause clinically significant distress or impairment in social, occupational, or other important areas of functioning; the episode is not attributable to the physiological effects of a substance or to another medical condition; and there has never been a manic episode or a hypomanic episode’ [31, 32]. Dysthymic Disorder is ‘characterized by a chronically depressed mood that occurs most of the day, more days than not, for at least 2 years’ [31, 32].

### Methodological assessment

We used the AIRE (Appraisal of Indicators through Research and Evaluation) instrument for the methodological assessment of the quality of the included articles [33]. It is a validated instrument that has been used previously in similar peer-reviewed studies [34–36]. The AIRE instrument contains 20 items, subdivided into four domains: (a) purpose, relevance and organizational context; (b) stakeholder involvement; (c) scientific evidence; and (d) additional evidence, formulation, and usage (see Additional file 2).

Two authors (YP and YS) independently appraised the included studies with the AIRE instrument. The AIRE instrument was applied to each completed set of quality indicators, because publications generally provided information about the development and scientific evidence of the total set of indicators instead of for each indicator separately [34–36]. Each item of the instrument has a score ranging from 1 to 4 with 1—strongly disagree (confident that the criterion has not been fulfilled or no information was available); 2/3—disagree/agree (unsure whether the criterion has been fulfilled); and 4—strongly agree (confident that the criterion has been fulfilled) [33].

Scores for each of the four domains were calculated by summing up all the scores of the individual items in a category and standardizing the total as a percentage of the maximum possible score for that domain. The maximum possible score for each domain was calculated by multiplying the maximum score per item (4) by the number of items in that domain (5, 3, 3, 9) and the number of appraisers (2). Similarly, the minimum possible score was calculated by using the minimum score per item (1).

The standardized category score is the total score per domain, minus the minimum possible score for that domain, divided by the maximum possible score, minus the minimum possible score * 100%. The standardized score ranges between 0 and 100%, and a score of 50% and higher indicates a higher methodological quality for each domain of the instrument [35]. We conducted and reported this study according to the Preferred Reporting Items for Systematic Review and Meta-Analysis (PRISMA) statement (see Additional file 3). This review has not been registered with PROSPERO database.

### Data extraction

A structured data extraction form was used to describe the selected studies with respect to the quality of depression care among adults over 18 years in primary care settings. The extraction information consisted of the title of the article; the publication date; summary of the indicator selection process; and description of indicators applicable to primary care for depression, including type, numerator and denominator of each quality indicator. The identified quality indicators were categorized according to Donabedian’s ‘structure-process-outcome’ framework [37, 38].

Because we were examining indicators for use in primary and ambulatory care settings, we used a conceptual framework for primary care developed by Hogg et al. [39] to further categorize structural indicators. This framework includes ‘structural’ and ‘performance’ domains. The structural domain is divided into three components, including (1) the healthcare system, defined as the policies, stakeholders and factors at the system level that can influence primary care organizations and providers, (2) the practice context, defined as the factors at the community level that can influence the organization of the practice and the delivery of care, and (3) organization of the practice, defined as the structures and processes at the practice level.

## Results

### Search results

**Fig. 1:**
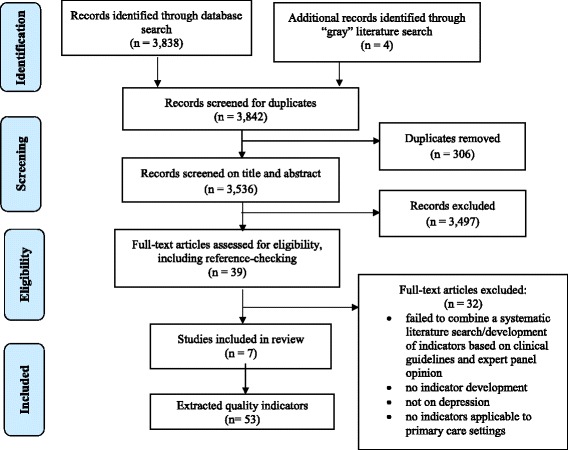
Flow diagram for selection of studies for the review

The systematic review identified 3838 potentially relevant studies from OVID MEDLINE and OVID PsycINFO (see Fig. 1). Four additional publications were identified through grey literature searching. After the review of titles/abstracts, 38 studies were deemed potentially relevant. One additional publication was included after tracking references. The full texts of these abstracts were obtained for review. Of these, 32 publications were excluded primarily because they failed to combine a systematic literature search or development of indicators based on clinical guidelines and expert panel opinion. Seven publications were included in the review (kappa = 0.91; very good agreement) [40].

### Study characteristics

The studies included in the review are summarized in Table 1. The majority of included studies were obtained from the USA or the UK. All included articles [41–47] used a combination of literature review/development of quality indicators from clinical guidelines and some form of consensus technique (Delphi, Modified Delphi methodology, or ‘constituency approach’) to derive a final set of quality indicators. Out of seven studies included in this review, only one study reported development of quality indicators using a clinical practice guideline. This guideline was the Veterans Health Administration Department of Defence (VHA/DoD) clinical practice guideline for the management of major depressive disorder in adults with further critical appraisal of indicators using a Delphi approach [43].

**Table 1:** Article characteristics

First author/organization	Organization/initiative	Country/year	Study design
Nakajima [41]	RAND/ACOVE	USA, 2007	Literature review for identifying candidate indicators; RAND/UCLA Appropriateness methodology for critical appraisal of indicators
Hermann [42]	–	USA, 2004	Literature review for identifying candidate indicators; Two-stage modified Delphi method for critical appraisal of indicators
Veterans Health Administration Department of Defence [43]	VHA/DOD	USA, 2000	Development of candidate indicators from guidelines; Delphi methodology for critical appraisal of indicators
Hermann [44]	OECD HCQI Project	OECD, 2006	Candidate indicators were drawn from OECD member countries quality initiatives; Modified Delphi methodology for critical appraisal of indicators
Canadian Mental Health Association (CMHA) [45]	CMHA, CEQM project	Canada, 2012	Literature review for identifying candidate indicators; 3-phase consensus-building methodology for critical appraisal of indicators
Shield [46]	–	UK, 2003	Literature review for identifying candidate indicators; Two-round Delphi methodology for critical appraisal of indicators
Worrall [47]	–	UK, 2002	Mix of literature review and stakeholder workshops for identifying candidate indicators; Consensus methodology for critical appraisal of indicators

*RAND* Research and Development, *ACOVE* Assessing the Care of Vulnerable Elders Project, *VHA*/*DOD* Veterans Health Administration Department of Defence, *OECD* Organisation for Economic Co-operation and Development, *CMHA* Canadian Mental Health Association

Included studies differed in terms of target population and settings, but all of them provided quality indicators applicable to primary care settings. One of the studies was focused on assessing care for vulnerable elders with major depression or dysthymia in both outpatient and inpatient settings [41]. Five studies [42, 44–47] aimed to develop a set of evidence-based indicators for mental health services, including those for depression and delivered within primary care settings for adults. Another study aimed to develop quality indicators for the management of major depressive disorders among adults in primary care settings [43].

### Methodological quality

The methodological quality of the included studies varied according to the AIRE instrument domains’ scores (Table 2). All studies were clear on the first AIRE instrument domain, demonstrating good evidence for describing the purpose of quality indicator development and the patient population to whom they were meant to apply, as well as presenting the indicator selection criteria and applicability of measures. Two studies [42, 43] received low scores for the second AIRE domain due to lack of information regarding the relevant stakeholders’ involvement at some stage of the indicator development process. The studies included in the present systematic review mainly represent the views of mental health administrators and clinicians, including psychiatrists and psychologists, as well as health service researchers and social workers. Only one included study represents patients’ perspectives on quality of depression care in developing quality indicators [47].

**Table 2:** AIRE instrument score

First author	AIRE instrument-standardized score (%)			
	Purpose, relevance and organizational context	Stakeholder involvement	Scientific evidence	Additional evidence, formulation and usage
Nakajima [41]	88	73	94	82
Hermann [42]	78	55	61	75
VHA/DOD [43]	73	54	55	78
Hermann [44]	78	83	60	72
CMHA [45]	76	72	61	54
Shield [46]	90	83	78	60
Worrall [47]	65	66	65	60

According to the Institute of Medicine (IOM), the appropriate high-level leadership, organization or experts, rather than those who developed the measure, should review and endorse measures of quality intended for population health improvement [48]. To this end, we identified which of the extracted quality indicators were endorsed by the National Quality Forum (NQF) and which were not. The National Quality Forum (NQF) is a voluntary not-for-profit membership organization created to develop and implement a national strategy for healthcare quality measurement and reporting. NQF evaluates measures against standardized measure evaluation criteria, including (1) importance to measure and report, (2) scientific acceptability of measure properties, (3) feasibility, (4) usability and use and (5) requirements for related and competing measures. NQF-endorsed consensus standards are now widely viewed as the ‘gold standard’ for measurement of healthcare quality, and NQF-endorsed measures are deemed to be evidence-based and valid [49].

The scientific evidence for the quality indicator development process was scarcely described in three articles [43–45]. Finally, most studies received high scores on the domain ‘Additional evidence, formulation, usage’; some initiatives reported numerator and denominator definitions, while others only provided the list of indicators. In the included studies, the quality indicators were appraised for different criteria, including importance of the quality indicators to be scientifically sound, valid, reliable and acceptable. Feasibility of data collection was assessed in four studies [42–44, 47]. In most studies, information regarding the piloting of indicators in practice and instructions for presenting and interpreting results were scarcely described.

### Quality indicators

Quality indicators were extracted only if they were relevant to the provision of primary care for depression. For the purpose of this study, the target population was defined as adults over 18 years with a diagnosis of major depression or dysthymia. The literature suggests that treatment of major depressive disorder also applies for dysthymia disorder [28–30, 50], and for the most part, quality indicators related to the management of major depression apply to that of dysthymia.

The identified quality indicators were organized according to Donabedian’s ‘structure-process-outcome’ framework [21, 38]. Structure indicators refer to settings where depression care is delivered, including adequate facilities, qualification of care providers or administration structure. Process indicators examine how depression care has been provided in terms of appropriateness, acceptability, completeness or competency. Outcome indicators refer to the end points of depression care, such as improvement in function or recovery [38].

A total of 53 quality indicators were identified from the included studies, many of which overlap conceptually or in content: 16 structure, 33 process and four outcome indicators (Table 3). Several quality indicators were represented in multiple articles; this might be a reflection of the attention to these areas of depression care in primary care settings.

**Table 3:** Quality indicators

Indicator	Source(s)	Description and/or numerator, denominator of indicator
Structure indicators		
Structural domain		
Governance and accountability	Shield, 2003 Worrall, 2002	Description: Written guidelines are in place to ensure that, where services are not provided locally, GPs can refer patients outside their locality (‘yes/no’ response).
	Shield, 2003 Worrall, 2002	Description: Specialist services are based on locally agreed written service plans and agreements which include the range, quality and volume of mental health services, including depression (‘yes/no’ response).
	Shield, 2003	Description: There is an agreed definition of depressive disorders which is explicit and standard within the practice (‘yes/no’ response).
	Shield, 2003	Description: There is a written complaints procedure which is prominently displayed regarding the provided care (‘yes/no’ response).
	Worrall, 2002	Description: There is a clear referral and feedback procedure for the practice counsellor (‘yes/no’ response).
Resources and technical provision	Shield, 2003 Worrall, 2002	Description: There is a demonstrable commitment to promote continuous professional and practice development in primary care (‘yes/no’ response).
	Shield, 2003 Worrall, 2002	Description: Practices are offered protected time for GPs and nurses to attend appropriate training courses (‘yes/no’ response).
Practice integration	Shield, 2003 Worrall, 2002	Description: There is a range of collaborative initiatives in place with other key agencies demonstrating effective partnerships (‘yes/no’ response).
Performance domain		
Access to care	Shield, 2003 Worrall, 2002	Description: Patients are able to make a routine appointment to see a general practitioner within 2 days (‘yes/no’ response).
	Shield, 2003 Worrall, 2002	Description: A member of the primary health care team is available as a point of contact for all patients to talk to in an emergency; clear written practice protocols are in place for obtaining specialist help in an emergency/crisis situation (‘yes/no’ response).
	Shield, 2003 Worrall, 2002	Description: There is equity of access to talking treatments regardless of ethnic origin, age, place of residence, socioeconomic status, and sex (‘yes/no’ response).
	Shield, 2003 Worrall, 2002	Description: There is good access to integrated and community-based mental health services out of hours, as well as locally agreed written standards and protocols for the delivery of out of hours care for mental health problems (‘yes/no’ response).
	Shield, 2003	Description: There is evidence of monitoring to ensure that out of hours standards are met (‘yes/no’ response).
Organizational structure and dynamics	Shield, 2003	Description: There are agreed written protocols and guidelines, based on best available evidence, for prescribing and monitoring psychotropic medication (‘yes/no’ response).
	Shield, 2003 Worrall, 2002	Description: Confidential discussions take place in private. There is an appropriate (i.e. private, quiet, relatively non-clinical) room for counselling/visiting mental health staff (‘yes/no’ response).
	Shield, 2003	Description: The confidentiality of medical records is protected and ensured at all times; where practicable, patient consent is sought before giving information to carers (‘yes/no’ response).
Process indicators		
Patient/caregiver education	Shield, 2003 CMHA, 2012 Worrall, 2002	Numerator: Number of patients from the denominator who received patient education at least once during the measurement period regarding depression, depression treatment, prescribed medication and coping strategies Denominator: Number of adult patients with a diagnosis of major depression or dysthymia during the measurement period.
Patient-provider relationship	Shield, 2003 Worrall, 2002	Description: Patients with depression are treated as individuals with individual needs and not as a ‘diagnosis of depression’. Treatment plans are individually tailored for each patient.
	Shield, 2003 Worrall, 2002	Description: Staff treats all patients with depression registered with the practice with respect, courtesy and consideration irrespective of age, sex, religious/cultural beliefs or diagnosis.
	Shield, 2003	Description: Staff are aware that patients with depression may be concerned about feelings of stigmatization and are treated in a way to minimize these feelings.
	Shield, 2003 Worrall, 2002	Description: Management time is available to support and lead change in service development; patients are not made to feel that they are wasting health professional’s time.
	Shield, 2003 Worrall, 2002	Description: Staff are aware of the potential impact of a depressive disorders on patient behaviour.
	Worrall, 2002	Description: Patient’s views about their condition are explicitly sought to help treatment adherence.
Shared decision-making	Shield, 2003 Worrall, 2002	Description: Patients are as fully involved as practicable in the formulation and delivery of their care and in any decisions about referral; where practicable, patients are informed of the reasons for referral to specialists or other professionals.
Up-to-date medical records	Shield, 2003	Description: Details of currently prescribed maintenance drugs are prominently recorded in the medical record. Medical records, including computerized records, are up to date and summarized.
Medication review	Shield, 2003	Numerator: Number of patients from the denominator who were on repeat maintenance drugs and offered regular reviews of their medication including monitoring for possible side effects and interactions with other drugs. Denominator: Number of adult patients with a diagnosis of major depression or dysthymia during the measurement period.
Depression comprehensive assessment/diagnosis	Shield, 2003 Worrall, 2002	Description: Physical symptoms in patients with depression are taken seriously and not automatically considered as psychosomatic; assessment takes into account language barriers, the needs of people with disabilities, ethnic, cultural and religious preferences.
	VHA/DOD, 2000 Hermann, 2004	Numerator: Number of patients from the denominator with a diagnosis of major depression or dysthymia during the previous 12 months. Denominator: Number of adult patients seen in a general medicine, primary care, women’s or mental health primary care clinic, during the previous 12 months.
Screening for and recognizing depression	Nakajima, 2007 Hermann, 2004 VHA/DOD, 2000 Shield, 2003 (NQF-endorsed)	Numerator: Number of patients from the denominator who were screened for depression using an age appropriate standardized tool and had follow-up plan documented, during the initial primary care evaluation and annually. Denominator: Number of adult patients seen in a general medicine, primary care, women’s or mental health primary care clinic, during the previous 12 months.
Documenting depression symptoms	Nakajima, 2007 Shield, 2003	Numerator: Number of patients from the denominator who have in the medical record at least three of the nine DSM-IV target symptoms for major depression were documented within 2 weeks of diagnosis. Denominator: Number of adult patients newly diagnosed with major depression or dysthymia during the measurement period.
Suicidal ideation	Nakajima, 2007 Hermann, 2004 Shield, 2003 (NQF-endorsed)	Numerator: Number of patients from the denominator who were assessed for suicidal ideation at initial evaluation. Denominator: Number of adult patients newly diagnosed with major depression or dysthymia during the measurement period.
Evaluate for comorbid conditions	Nakajima, 2007	Numerator: Number of patients from the denominator who had been evaluated for substance dependence or abuse for men, and hypothyroidism for women, within 1 month or in the prior 3 months. Denominator: Number of adult patients who were diagnosed with a new episode of major depression or dysthymia during the measurement period.
Initiating depression treatment	Nakajima, 2007 Shield, 2003	Numerator: Number of patients from the denominator who were offered antidepressant treatment, psychotherapy or electroconvulsive therapy within 2 weeks after diagnosis. Denominator: Number of adult patients who diagnosed with a new episode of major depression or dysthymia during the measurement period.
Treatment/monitoring	Shield, 2003	Description: No drug is prescribed unless the health professional understands the potential efficacy and side effects; prescribing for depression is based on up to date evidence and, where available, local management protocols.
	Shield, 2003	Numerator: Number of patients from the denominator who were not responding to first line drug treatment at the therapeutic dosage and were asked about adherence. Denominator: Number of adult patients with a diagnosis of major depression or dysthymia during the measurement period.
	Shield, 2003	Numerator: Number of patients from the denominator who were experiencing difficulties undertaking withdrawal from medication and were offered referral to a mental health worker. Denominator: Number of adult patients with a diagnosis of major depression or dysthymia during the measurement period.
Antidepressant choice	Nakajima, 2007 (‘Negative indicator’)	Numerator: Number of patients from the denominator who were prescribed antidepressants using tertiary amine tricyclics, MAOIs (unless atypical depression is present), benzodiazepines or stimulants (except methylphenidate) as first- or second-line therapy. Denominator: Number of adult patients with a diagnosis of major depression or dysthymia during the measurement period.
	Hermann, 2006 Hermann, 2004 (‘Negative indicator’)	Numerator: Number of patients from the denominator who were prescribed anticholinergic antidepressants as first- or second-line therapy. Denominator: Number of patients aged 65 and older with a diagnosis of major depression or dysthymia during the measurement period.
	Shield, 2003	Description: Choice of medication is based on individual patient factors including the desirability of sedation, previous response to a drug treatment including adverse reactions, co-morbid psychiatric or medical conditions, concurrent drug treatment and relative risk of medication in overdose.
Interactions with monoamine oxidase inhibitor (MAOI)	Nakajima, 2007	Description: If a patient with a diagnosis of major depression or dysthymia is taking an selective serotonin reuptake inhibitor (SSRI), then an MAOI should not be used for at least 2 weeks after termination of the SSRI and vice versa.
Continuing antidepressant medication treatment in acute phase	Hermann, 2004 Hermann, 2006 VHA/DOD, 2000 (NQF-endorsed)	Numerator: Number of patients from the denominator who responded to antidepressant medication and remained on an antidepressant treatment for at least 3 months (12 weeks). Denominator: Number of adult patients who diagnosed with a new episode of depression or dysthymia during the measurement period.
	Nakajima, 2007 CMHA, 2012	Numerator: Number of patients from the denominator who had no meaningful symptom response after 6 weeks of psychotherapy treatment (without medication) and for whom the medication treatment has been initiated, a patients was referred to a psychiatrist by the 8th week of depression treatment. Denominator: Number of adult patients who diagnosed with a new episode of major depression or dysthymia, during the measurement period.
	Nakajima, 2007 CMHA, 2012	Numerator: Number of patients from the denominator who had no meaningful symptom response after 6 weeks of drug treatment and the drug dose was optimized or changed, or a patient was referred to a psychiatrist by the 8th week of depression treatment. Denominator: Number of adult patients who diagnosed with a new episode of major depression or dysthymia, during the measurement period.
Continuing depression therapy in continuation phase	Nakajima, 2007 Hermann, 2006 Hermann, 2004 (NQF-endorsed)	Numerator: Number of patients from the denominator who responded to antidepressant medication, remained on the drug at the same dose for at least 6 months. Denominator: Number of adult patients newly diagnosed with and treated for major depression or dysthymia, during the measurement period.
	Nakajima, 2007 CMHA, 2012	Numerator: Number of patients from the denominator who experienced three or more episodes of depression and received maintenance antidepressant medication with the same type and dose of medication for at least 24 months, with at least four office or telephone visits for depression during that period. Denominator: Number of adult patients newly diagnosed with and treated for major depression or dysthymia during the measurement period.
Effectiveness	VHA/DOD, 2000 CMHA, 2012	Numerator: Number of patients from the denominator who had a systematic symptom assessment at 12 weeks following diagnosis, or if in remission by week 12, then a systematic symptom assessment is performed at the time of remission. Denominator: Number of adult patients newly diagnosed with and treated for major depression or dysthymia during a 12-month period.
Psychotic depression treatment	Nakajima, 2007 Hermann, 2004	Numerator: Number of patients from the denominator who had been referred to a psychiatrist or received treatment with a combination of an antidepressant and an antipsychotic. Denominator: Number of adult patients with a diagnosis of major depression with psychotic features, during the measurement period.
Visits during acute phase treatment of depression	Hermann, 2006 Hermann, 2004	Numerator: Number of patients from the denominator who received at least three medication visits or at least eight psychotherapy visits in a 12-week period. Denominator: Number of adult patients who diagnosed with a new episode of major depression or dysthymia, during the measurement period.
Depression follow-up	Shield, 2003 CMHA, 2012	Description: Patients with severe depression are offered regular appointments to monitor and follow up treatment, symptoms, side effects and adherence.
Outcome indicators		
Depression remission at 6 months	National Quality Measures Clearinghouse (NQMC) (NQF-endorsed)	Numerator: Number of patients from the denominator with an initial PHQ-9 score greater than nine who achieve remission at 6 months as demonstrated by a 6-month (±30 days) PHQ-9 score of less than five. Denominator: Number of adult patients with a diagnosis of major depression or dysthymia and an initial PHQ-9 score greater than nine, during the measurement period.
Depression re-emission at 12 months	National Quality Measures Clearinghouse (NQMC) (NQF-endorsed)	Numerator: Number of patients from the denominator with an initial PHQ-9 score greater than nine who achieve remission at 12 months as demonstrated by a 12-month (±30 days) PHQ-9 score of less than five. Denominator: Number of adult patients with a diagnosis of major depression or dysthymia and an initial PHQ-9 score greater than nine, during the measurement period.
Depression response at 6-month progress towards remission	National Quality Measures Clearinghouse (NQMC) (NQF-endorsed)	Numerator: Number of patients from the denominator with an initial PHQ-9 score greater than nine who achieve a response at 6 months as demonstrated by a 6-month (±30 days) PHQ-9 score that is reduced by 50% or greater from the initial PHQ-9 score. Denominator: Number of adult patients with a diagnosis of major depression or dysthymia and an initial PHQ-9 score greater than nine, during the measurement period.
Depression response at 12-month progress towards remission	National Quality Measures Clearinghouse (NQMC) (NQF-endorsed)	Numerator: Number of patients from the denominator with an initial PHQ-9 score greater than nine who achieve a response at 12 months as demonstrated by a 12-month (±30 days) PHQ-9 score that is reduced by 50% or greater from the initial PHQ-9 score. Denominator: Number of adult patients with a diagnosis of major depression or dysthymia and an initial PHQ-9 score greater than nine.

The structure indicators were derived from two articles that aimed to develop indicators to evaluate primary care services for people with depression [46, 47]. They were categorized into two main domains: (1) structural, including quality improvement process, resources and technical provisions, and practice integration and (2) performance, including access to care, and organizational structure and dynamics [39].

Process indicators represent the way depression care is delivered, encompassing both clinical effectiveness and interpersonal effectiveness. The identified process indicators have been categorized into the following common groups: depression screening and detection; screening for suicidal ideation; assessment for comorbid conditions; initiating, continuing and monitoring depression treatment; antidepressant choice; and depression follow-up, as well as patient/caregiver education, patient-provider relationship and shared decision-making. We identified a few ‘negative’ or ‘do not do’ process indicators, including the use of anticholinergic antidepressants, tertiary amine tricyclics, MAO inhibitors or benzodiazepines as first- or second-line therapy in older adults with depression [41, 42, 44].

The outcome indicators identified in this study have been developed by the National Quality Measures Clearinghouse (NQMC) and published by the Agency for Healthcare Research and Quality (AHRQ) [51–54] and endorsed by the National Quality Forum (NQF). They focused on response to treatment and achieving remission at 6 or 12 months of depression treatment.

## Discussion and conclusions

The current systematic review identified 53 quality indicators for evaluating primary care for depression. They assess multiple aspects of primary care for depression, including organizational and clinical aspects of depression care, as well as policy importance. All included studies used a rigorous method of developing quality indicators [11, 25, 26] by combining a systematic literature search with appraisal of candidate indicators using expert panel opinion. Despite the importance of depression care, relatively few of the identified quality indicators are formally endorsed as legitimate measures of quality of depression care.

One of the important aspects when developing indicators is involvement of different stakeholders with different perspectives on quality of care. The studies included in the present systematic review mainly represent the views of mental health administrators and clinicians, including psychiatrists and psychologists, as well as health service researchers and social workers. Only one included study represented patients’ perspectives on quality of depression care in developing quality indicators [47]. In the process of developing quality indicators, it is recommended to include the perspectives of all potential end users including patients, their family caregivers, health professionals and managers [55]. There is a particular need to ensure that indicators reflect what matters most to patients.

Most quality indicators identified in this study focused on the structure or process of primary care for depression. The identified structure indicators have been categorized into structural and performance domains. The identified process indicators measure the activities and tasks in primary care for depression, including health providers’ activities in screening and detecting depression and initiating, implementing and monitoring depression treatment, as well as interacting with patients. We identified a few ‘negative’ or ‘do not use’ indicators, including the use of anticholinergic or tetracyclic antidepressants, benzodiazepines and MAO inhibitors as first- or second-line therapy in older adults with depression.

Outcome indicators identified in this study related to the reduction in the number and severity of depressive symptoms that serve as a marker of wellness of patients with depression. We observed that a limited number of outcome indicators were developed for evaluation of care for depression. Overall, reasons for the small number of outcome indicators may be the limited scientific evidence linking structure and process to outcomes of depression care or perhaps the length of time it takes to assess outcomes due to the often long-term and fluctuating nature of depression or lack of understanding of what meaningful outcomes could/should be measured.

We identified a comprehensive list of quality indicators, including structure, process and outcome indicators. Mainz et al. stated that, although the healthcare providers might need detailed information about the process of depression care for quality improvement purposes, payers of the care and consumers might be more interested in structure and outcomes of the care [20]. Therefore, a combination of structure, process and outcome indicators might be most suitable for measuring the quality of care for depression in primary care settings, and this study provides a useful framework for continued use as this field presumably grows.

Despite the growing interest in assessing the quality of care for depression, there has been little evaluation of the quality of care for depression in primary care settings [11, 24]. For instance, Duhoux and colleagues [24] performed a systematic review of quality indicators for depression treatment in primary care. The authors of this study aimed to systematically review indicators for measuring the quality of depression treatment in primary care related to pharmacotherapy, psychotherapy or educating patients about depression.

They found that most studies used rudimentary indicators to measure the quality of depression treatment, the studies differed greatly with respect to the methods used and most studies revealed that a large proportion of people with depression do not receive minimally adequate treatment in primary care settings [24]. In contrast to the previous studies, the main strength of the present systematic review is that all included studies developed quality indicators using rigorous methods, by combining a systematic literature search/developing indicators from clinical guidelines with appraisal of candidate indicators using expert panel opinion.

Overall, the present systematic review revealed poor reporting of the methods used for developing quality indicators that complicated assessment of the methodological rigour and quality of the studies.

The assessment and monitoring of care quality can be achieved by using quality indicators which are based on standards of care and the best available evidence [20]. Data generated from these measures can be used to assess past performance, identify suboptimal practices and plan improvements. To this end, studies on development of quality indicators should be periodically updated to align with current standards of care. The literature suggests that quality indicators cannot be transferred from one country to another without a prior validation, and often translation, to take into account variations in clinical practice and professional culture [56–58].

The majority of included studies were obtained from the USA or the UK. When selecting healthcare indicators to be used locally, it is important to ensure that they reflect local circumstances and that they can be used to develop local standards of care [56]. In this way, differences in policy priorities, the organization of healthcare system and clinical practice can be addressed.

The feasibility of indicator measurement is another important consideration. It was identified that indicators were assessed in the studies included in this review. Quality indicators identified in this review utilized three common sources of data: secondary data (administrative databases), clinical data (medical records) and survey data.

It is well established that depression is associated with a wide range of chronic conditions, including diabetes, cardiovascular disease, cancer and arthritis, and that it has a negative impact on the outcomes of these conditions [8–10]. However, the methodology to measure quality of care for diabetes patients with multiple chronic conditions has been poorly developed. Therefore, a multidisciplinary expert panel was recruited from across Canada to critically appraise the resulted quality indicators and select the appropriate set of quality indicators for evaluating care for patients with depression comorbid with other chronic conditions. It will be reported in a separate manuscript.

### Limitations

As demonstrated in this study, relatively little rigorous research has been done to develop quality indicators to assess the quality of primary care for depression. However, our intention to include only indicators that have been developed through an evidence-based approach, including a combination of literature search with expert panel opinion, may have led to the exclusion of some indicators that were developed using other approaches. Our literature search was restricted to studies published in English that omits potentially relevant publications in other languages.

The AIRE instrument that was used to assess the quality of the included studies is mainly focused on the indicator development process. We may underestimate the methodological quality of some studies, because the information related to the indicator development process was not always described within the articles. We tried to track down additional information in the literature about the development process of quality indicators, but we were able to get relevant additional information only for three sets of quality indicators. Due to time constraints, we did not contact any organization/author to elicit any additional information. The study results indicate the need for further development of quality indicators with detailed methodological specifications for monitoring and accurate assessment of the care for adults with depression in primary care settings.

Overall, we conclude that evidence-based and valid quality indicators for assessing quality of primary care for depression are scarce, but the identified set of indicators address multiple dimensions of depression care and provide an excellent starting point for further development. As the disease burden of depression is high, and much of it is presented clinically to general practitioners, incorporation of these indicators to routine primary care practice is recommended. Periodic evaluation reports of primary care for depression can be useful to monitor performance and serve to evaluate effectiveness of depression care among a general adult population.

Quality indicators should be valid and sensitive to the changes they are intended to detect and should be linked to improving patient outcomes [20]. There is a need to develop specific structure, process and outcome measures for people with depression by engaging clinicians, patients and families in the identification of meaningful measures and then determining how they could be collected systematically. Future research is required to implement the identified set of quality indicators in this study and to examine the association between identified structures and processes and depression care outcomes.

## Additional files

Additional file 1: Search strategies. (DOCX 31 kb)

Additional file 2: AIRE instrument. (DOCX 30 kb)

Additional file 3: PRISMA checklist. (PDF 176 kb)

## Abbreviations

AIRE: Appraisal of Indicators through Research and Evaluation
NQMC: National Quality Measures Clearinghouse
NQF: National Quality Forum
RAND Health Corporation: Research and Development Health Corporation
ACOVE: Assessing Care of Vulnerable Elders
VHA/DOD: Veterans Health Administration Department of Defence
OECD: Organisation for Economic Co-operation and Development
CMHA: Canadian Mental Health Association
AHRQ: Agency for Healthcare Research and Quality
MAOIs: Monoamine oxidase inhibitors
