# Supplementary material for: Quality indicators for care of depression in primary care settings: a systematic review
Source: Syst Rev. 2017 Jul 3;6:126. doi: 10.1186/s13643-017-0530-7 (PMC5496323; doi:10.1186/s13643-017-0530-7)
Supplement: Supplementary file 1 — Search strategies. (DOCX 31 kb) [file 13643_2017_530_MOESM1_ESM.docx]

**Additional file 1. OVID MEDLINE search strategies**

Ovid MEDLINE(R), Ovid MEDLINE(R) In-Process

| # | Searches |
| --- | --- |
| 1 | quality of health care/ or guidelines as topic/ or exp quality assurance, health care/ or benchmarking/ or report card/ or exp quality indicators, health care/ or exp "outcome and process assessment (health care)"/ or ((quality adj2 (healthcare or health care)) or ((("health care" or healthcare) adj3 benchmarking) or (best adj practice adj analysis) or benchmark$) or ((quality adj3 indicator? adj3 healthcare) or (quality adj2 indicator) or (indicator$ adj measure$)) or ((measure? or assessment?) adj4 (outcome or process))).ti,ab. |
| 2 | exp Depression/di, dt, mo, pc, th [Diagnosis, Drug Therapy, Mortality, Prevention & Control, Therapy] |
| 3 | exp Depressive Disorder/di, dt, mo, pc, th [Diagnosis, Drug Therapy, Mortality, Prevention & Control, Therapy] |
| 4 | 2 or 3 |
| 5 | 1 and 3 |
| 6 | limit 5 to (english language and humans and yr="2000 - current" and ("all adult (19 plus years)) |
